# Neural Mechanisms of Cognitive Behavioral Therapy Efficacy in Anxiety Disorders: A Scoping Review of fMRI-Based Studies That Tested the Dual Model

**DOI:** 10.3390/life15030493

**Published:** 2025-03-18

**Authors:** Judith Domínguez-Pérez, Wenceslao Peñate-Castro, Francisco Luis Rivero-Pérez

**Affiliations:** 1 Department of Clinical Psychology, Psychobiology and Methodology, Universidad de La Laguna, Campus de Guajara, 38200 La Laguna, Spain; jdomper.00@ull.edu.es; 2 Departament of Psychology, Universidad Europea de Canarias, 38300 La Orotava, Spain; franciscoluis.rivero@universidadeuropea.es

**Keywords:** cognitive behavioral therapy, anxiety disorders, specific phobia, dual-route model

## Abstract

Anxiety disorders are common mental health conditions characterized by excessive fear and anxiety. Cognitive behavioral therapy (CBT) has demonstrated efficacy, yet the possible neurobiological mechanisms underpinning its effects remain debated. The dual-route model offers a potential explanation by suggesting that CBT increases activation in the brain areas responsible for emotion regulation while decreasing activation in limbic areas. This scoping review examined possible neurobiological mechanisms supporting CBT’s efficacy in anxiety disorders by exclusively reviewing studies that utilized functional magnetic resonance imaging (fMRI). The included studies published since 2018 focused on adult samples that received CBT for anxiety disorders, with pre- or post-treatment fMRI data. The data extraction followed a standardized process, with key variables, such as the study design, population, and neuroimaging findings, systematically charted. While the dual-route model explains key aspects of CBT’s neurobiological effects, the findings suggest the need for expansion to incorporate areas such as the precuneus, particularly in specific phobias and social anxiety disorder. Further research is required to refine this model and explore additional mechanisms underlying CBT’s efficacy.

## 1. Introduction

Anxiety disorders are mental health conditions characterized by excessive fear, anxiety, and associated behavioral disturbances. The symptoms of anxiety disorders lead to significant distress or impairment in personal, familial, social, educational, occupational, or other essential areas of life. This category includes generalized anxiety disorder, panic disorder, social anxiety disorder, specific phobias, agoraphobia, separation anxiety disorder, and selective mutism [1]. These disorders share common features of excessive fear and anxiety, both of which manifest through heightened physiological responses, cognitive distortions, and avoidance behaviors. Fear is defined as an emotional response to an imminent, real, or imagined threat, meanwhile anxiety is the anticipatory response to a future threat. These conditions affect a substantial portion of the population, significantly reducing their quality of life and interfering with personal, social, and occupational functioning [2,3].

Currently, several treatments are considered effective at managing these disorders [4,5], including gradual exposure, cognitive behavioral therapy (CBT), acceptance and commitment therapy (ACT), or mindfulness. However, an ongoing challenge in psychotherapy research is identifying the specific mechanisms underlying their effectiveness. Interestingly, despite their divergent conceptual foundations, different psychotherapeutic methods are effective at treating the same mental health disorders, including anxiety disorders [6].

One approach to investigate the underlying mechanisms of psychotherapy is through a neuroscientific perspective. Changes in psychological states are often reflected in neural connectivity modifications [7]. These changes may extend to structural changes in the brain. Consequently, there has been a preference for experimental studies on the brain activity of individuals receiving different psychological treatments. Initial neurocognitive research has provided evidence consistent with the theoretical model of CBT: if cognitive work generates positive effects, the processing of anxiety contexts would be characterized by a greater activation of executive areas responsible for emotional regulation (such as the ventromedial and dorsolateral prefrontal cortex) and, consequently, a decrease in the activation of limbic areas, especially the amygdala. This phenomenon has been conceptualized as the dual-route model [8]. Essentially, the dual-route model describes a balance between an automatic, impulsive fear-processing response (impulsive) and a top-down, cognitive regulatory response (reflective), which is observable in the described changes. Therefore, the dual-route model provides a framework to explain how CBT influences brain function in anxiety disorders. According to this model, two opposing neural routes regulate anxiety:

1. The impulsive route, dominated by the amygdala and other limbic structures, drives automatic fear responses.
2. The reflective route, primarily involving the prefrontal cortex (e.g., ventromedial and dorsolateral prefrontal cortex), modulates emotional responses through cognitive regulation.

**Figure 1:**
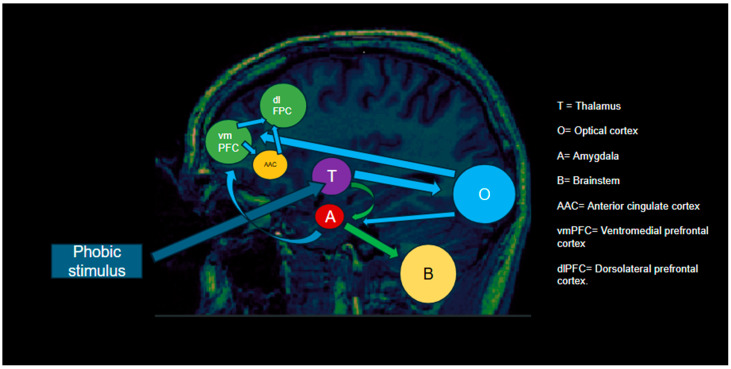
Schematic representation of the dual-route model in anxiety disorders. The impulsive route (green arrows) rapidly processes fear-related stimuli via the amygdala, triggering an automatic fear response. The reflective route (blue arrows) engages prefrontal regions to regulate amygdala activity, promoting cognitive control over fear reactions.

In the Figure 1, a more detailed schematic representation can be observed. In this representation, green arrows signify the impulsive route and blue arrows correspond to the reflective route.

– Impulsive route:
  1. A phobic stimulus (e.g., a spider) is seen.
  2. The thalamus receives information for sensorial processing (T).
  3. Information reaches the amygdala with fear activation (A).
  4. The amygdala sends information to the brainstem, activating the fight/flight response (B).

– Reflective route:
  1. After the thalamus has received the information, it is sent to the visual cortex.
  2. The visual cortex (O) processes the stimulus in detail and sends the information to the ventromedial prefrontal cortex (vmPFC) and the amygdala.
  3. The amygdala sends the information to the vmPFC as well.
  4. The vmPFC and the anterior cingulate cortex (ACC) assess the real danger and send the information to the dorsolateral prefrontal cortex (dlPFC).
  5. The dlPFC provides inhibitory control to the amygdala, modulating the fear response.

CBT is hypothesized to enhance the reflective route, leading to greater control over emotional responses and a reduction in limbic hyperactivation. Early functional magnetic resonance imaging (fMRI) studies support this model, showing that after CBT, individuals exhibited increased activation of the ventromedial and dorsolateral prefrontal cortex and decreased activation of the amygdala [9,10].

Nonetheless, not all available data fully align with the dual-route model. Some studies report that prefrontal activation does not always increase following CBT [8] (p. 2). Other studies suggest that there is lower activation in these areas and greater activation in other associative areas, such as the anterior cingulate gyrus and superior parietal areas [11].

### Objectives

Given the growing body of research questioning the completeness of the dual-route model, this study aimed to provide a comprehensive review of its applicability in explaining CBT’s neurobiological effects in anxiety disorders. Specifically, this scoping review sought to achieve the following:

– Assess the validity of the dual-route model in explaining CBT’s efficacy for specific phobias.
– Examine whether this model applies to broader anxiety disorders, particularly social anxiety disorder and panic disorder.
– Identify additional neural mechanisms beyond the dual-route model that contribute to CBT efficacy, focusing on precuneus involvement and other associative regions.
– By mapping the available literature, this review contributes to refining current neurobiological models of CBT, potentially expanding the dual-route model to better reflect the complexity of neural adaptations in anxiety treatment.

## 2. Materials and Methods

To achieve these objectives, a review was conducted following the Preferred Reporting Items for Systematic Reviews and Meta-Analyses extension for Scoping Reviews (PRISMA-ScR) Checklist, a 22-item checklist designed to promote transparency by explaining the rationale for this review, the methods used, and the results obtained [12].

### 2.1. Protocol and Registration

This scoping review has been registered on 16 November 2024, in the OSF under the title CBT Effectiveness on Anxiety Disorders, with the following registration DOI: https://doi.org/10.17605/OSF.IO/2JEVP.

### 2.2. Eligibility Criteria

Inclusion criteria: Studies needed to meet the following criteria:

- Studies published from 2018 to November 2024 were included. To ensure that this review encompassed the most recent advancements in cognitive behavioral therapy (CBT) and functional magnetic resonance imaging (fMRI), and given the significant contributions by Lueken and Hahn (2016) [8] (p. 2), who made notable advancements in understanding the dual-route model of CBT for anxiety disorders, this year was selected as the starting point for our search. The starting point was set to 2018 to ensure that this review incorporated studies that built upon key advancements in understanding the dual-route model, particularly those proposed by Lueken and Hahn (2016) [8] (p. 2). Given the time required for scientific findings to influence subsequent research, we selected this timeframe to capture studies that applied and expanded upon these foundational concepts while reflecting methodological improvements in neuroimaging research, with the aim to incorporate the most current evidence related to the dual-route model and its application. Earlier studies, while valuable, often utilized different imaging techniques and analytical frameworks that may not align with the scope of this review.
- Experimental studies, randomized clinical trials, or randomized controlled trials that applied some CBT procedure.
- CBT was used as a treatment for phobic and/or anxiety disorders with clinical or subclinical samples.
- Studies that provided improvement in phobic and/or anxiety symptoms as a consequence of CBT application.
- Studies that provided neuroimage data about CBT efficacy.
- Studies that used adult samples.
- Studies published in Spanish, English, or French.

Exclusion criteria:

- Observational studies (cohorts, cases/non-cases, cross-sectionals), single-case designs, qualitative studies, or literature reviews.
- Studies where phobic and/or anxiety disorders were secondary disorders to a major disorder.
- Studies that did not provide fMRI data.
- The fMRI data provided could not test the presence or absence of the dual-route model.

### 2.3. Information Sources

The review search was conducted using the following databases: SCOPUS, APA PsycInfo, APA PsycArticles, Web of Science, and SpringerLink.

### 2.4. Search Strategy

All inclusion criteria needed to be simultaneously met (AND logic applied). Therefore, the search string used was as follows: (“anxiety disorders” OR “Generalized Anxiety Disorder” OR “specific phobia” OR “Agoraphobia” OR “Panic Disorder” OR “Social Anxiety Disorder”) AND (“cognitive behavioral therapy” OR “CBT” OR “cognitive therapy” OR “behavioral therapy” OR “exposure therapy”) AND (“functional magnetic resonance imaging” OR “fMRI” OR “neuroimaging” OR “brain imaging” OR “functional MRI”).

### 2.5. Selection of Sources of Evidence

Only peer-reviewed scientific articles were considered for inclusion in this scoping review. Other sources, such as conference proceedings, symposium abstracts, or unpublished data, were excluded to ensure the methodological rigor and reliability of the findings.

The selection process adhered to the PRISMA flow diagram. First, in the identification phase, duplicates were removed. During the screening phase, titles and abstracts were revised, and publications that did not meet the inclusion and exclusion criteria were eliminated. Afterward, publications selected for the eligibility assessment were read in full, with those deemed ineligible being excluded. Finally, studies that met all the inclusion criteria were included in the systematic review.

Additionally, the Cochrane quality assessment scale was used to evaluate the methodological quality of the included studies. To ensure methodological rigor, in compliance with the Cochrane quality assessment, the studies with a high risk of bias were excluded according to the following exclusion criteria:

- Small sample size (*n* < 15).
- Absence of clear randomization procedures.
- Incomplete reporting of dropout rates.
- Incomplete reporting of data.

No articles were excluded for risk of bias.

### 2.6. Data-Charting Process

The data-charting process was conducted using a predefined and calibrated form to extract key information systematically from each included study. Charting was performed independently by two reviewers to enhance the reliability, with discrepancies resolved through discussion or consultation with a third reviewer. This ensured consistency in capturing the relevant variables, such as the study design, population characteristics, intervention details, and key outcomes.

### 2.7. Data Items

The following key data items were charted from the included studies and structured into a summary table:

- Author(s): the primary authors of each study.
- Disorder: the specific anxiety disorder addressed in the study.
- Participants: sample size and characteristics (e.g., patient vs. control groups).
- Results: key findings, particularly those related to neuroimaging data (e.g., changes in the brain activity pre- and post-CBT).

This approach ensured that the data extraction process aligned directly with the scoping review objectives to facilitate a clear synthesis of the findings.

### 2.8. Critical Appraisal of Individual Sources

The quality of the included studies was assessed using the Cochrane quality assessment scale [13], a standardized tool for evaluating methodological rigor. This appraisal considered elements such as randomization, blinding, the control of confounding factors, and reporting completeness. The results of this evaluation were used to contextualize the findings and identify potential biases within and varying levels of evidence quality between the included studies.

### 2.9. Synthesis of Results

The synthesis of results was performed narratively by summarizing the charted data in relation to the review objectives. Patterns in neuroimaging findings were identified and linked to the theoretical framework of the dual-route model. Discrepancies between studies, such as differences in the prefrontal cortex activation, were highlighted to reflect the variability in the evidence. The results were tabulated to present key findings systematically, while the narrative discussion emphasized overarching trends, gaps in the knowledge, and potential implications for future research.

## 3. Results

**Figure 2:**
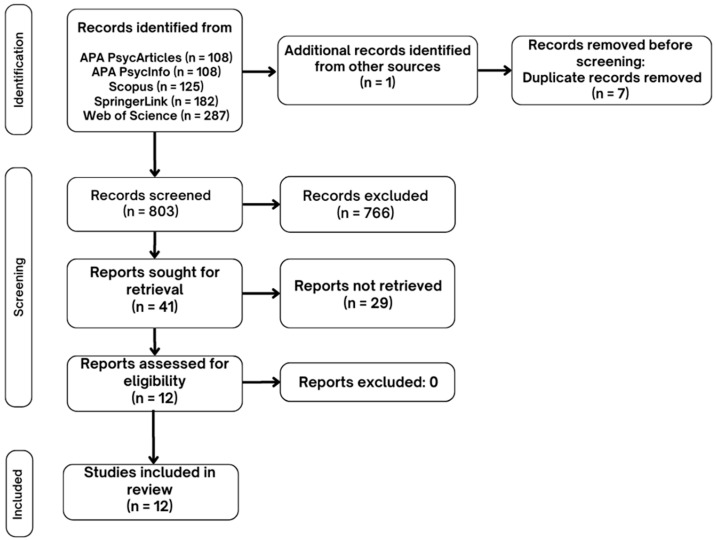
Flowchart of the search, identification, and final selection of references included in this review.

The selection and analysis process for the studies in this scoping review followed the PRISMA flow diagram. In Figure 2, the selection process is summarized. Initially, 803 studies were identified in specific databases after removing duplicates. Based on the inclusion and exclusion criteria, 12 studies were finally selected and included in this scoping review.

**Figure 3:**
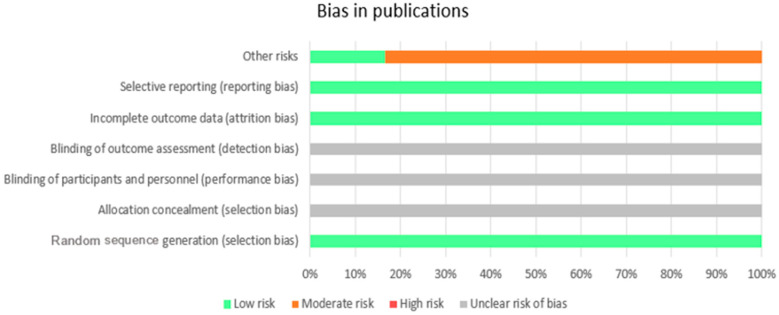
Chart of global bias in publications.

Regarding the bias risk of the articles, the Cochrane quality assessment scale was administered to assess their quality. In Figure 3, the chart presented provides a visual representation of the bias levels in different categories, indicating areas of low, moderate, high, and unclear risks. As can be observed, almost all publications complied with the reporting dropout rates and informed about the potential methodological limitations of their studies. Half of the studies reported on the randomization process. The highest risks were identified in the detection of other risks (for example, having a small sample, not having a control group or patients being medicated for their disorder). Additionally, the aspect that was expressed with the least clarity was blinding and allocation concealment.

**Figure 4:**
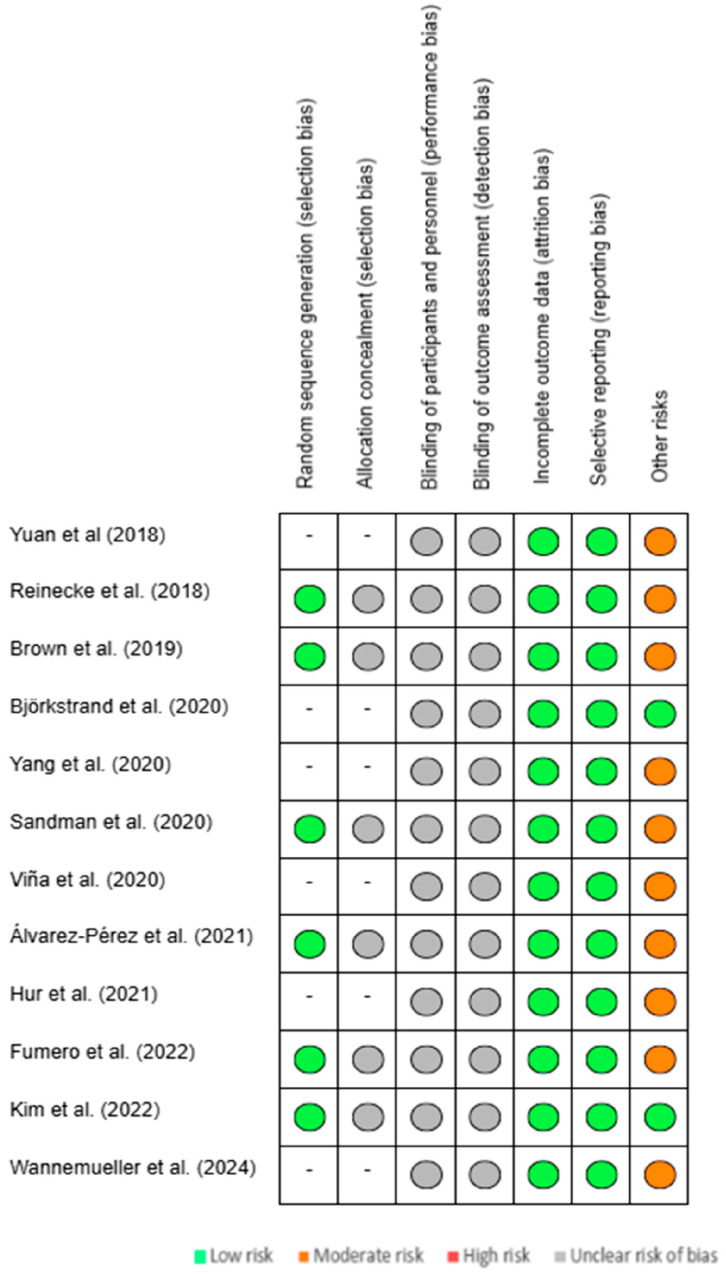
Chart of bias risk for each publication included [14,15,16,17,18,19,20,21,22,23,24,25].

In Figure 4, the chart indicates the level of bias identified in each study, classifying the risks as low, moderate, high, or unclear in the different categories mentioned above.

In Table 1, there is a summary of the main results found in each included study, as well as the authors, the disorder that was analyzed, and the participants involved.

A summary of the quantity of studies for each disorder can be found in Table 2. It can be observed that specific phobias were the disorders that had more articles, followed by social anxiety disorder and, finally, panic disorder.

In Table 3, Table 4 and Table 5, a summary of the brain regions and their activation underlying CBT can be found according to each disorder and taking into account the dual-route-model-related areas.

## 4. Discussion

In this scoping review, the main objective was to explore the factors contributing to the efficacy of CBT in specific phobias and to provide evidence to clarify or expand the ongoing debate surrounding the dual-route model, which currently explains CBT’s effectiveness in this disorder. A secondary objective was to explore whether the mechanisms of the dual-route model also apply to anxiety disorders more broadly, providing evidence for CBT’s efficacy in treating them.

### 4.1. Specific Phobias

Across all the studies, the baseline neuroimaging data indicate hyperactivation of the amygdala, anterior cingulate cortex (ACC), and insula before the CBT treatment, aligning with the dominance of automatic fear responses predicted by the dual-route model. CBT consistently reduces subcortical activity and improves top-down regulation, restoring the balance between the cortical and subcortical systems. Although the model predicts a significant reduction in subcortical activity, some studies show residual responses in the amygdala even after treatment [21], suggesting that cortical regulation may not be entirely effective in all cases. According to this result, and the fact that in all studies, the subjective anxiety levels were reduced, and therefore, CBT worked, there is a need to explore the reasons behind this. Some studies emphasized intermediate areas, such as the lateral parietal cortex or the precuneus, which are not in the traditional dual-route model. The precuneus seems to be a key node in anxiety disorders and, more specifically, in specific phobias, as it is involved in self-referential processing, plays an important role in emotional processing, and shows improvements following treatment. Additionally, the precuneus activity seems to be different between phobic stimuli after treatment. Small-animal phobias were related to enhanced precuneus activity after CBT [20,21,23], which can be related to better emotional regulation and the ability to reorganize phobic stimuli meanings. On the other hand, the dental phobia experiment [25] found a reduction in the precuneus activation, which can be related to reduced egocentric processing or rumination over phobic stimuli.

### 4.2. Social Anxiety Disorder

The amygdala, insula, and prefrontal areas were consistently affected after CBT regarding clinical improvement. Other areas seem relevant for CBT’s effectiveness, such as the precuneus, which was proved to have reduced activation after the treatment [14] and seemed to be altered before the treatment [16,19,24]. Although these studies did not mention what happened to the precuneus activation post-treatment, it is implied that as a central aspect in negative auto-referential processes and rumination, the reduction in its hyperactivity leads to a disconnection in persistent negative thoughts and improvement in emotional regulation.

Fusiform gyrus activation showed a reduction in its activation as well [16,24], which implies a normalization in the reactivity and sensitivity to visual information during social evaluation. The medial temporal gyrus also shows enhanced connectivity after CBT [14,22], which provides a better ability to integrate sensory and linguistic information in social contexts. The posterior cingulate cortex also shows reduced activation after treatment, which may indicate a reduction in negative thoughts.

The dual-route model, which is understood as a balance between hyperactivation and regulation, can be extrapolated to SAD. However, the traditional areas involved need to be expanded, including the default mode network and sensorial areas that contribute to the disorder, as extracted from the results.

### 4.3. Panic Disorder

The results of the panic disorder studies show that there was a reduction in amygdala activation, which was related to clinical improvement. Although ACC was heavily studied by Yang et al. (2020) [18] in comparison with Reinecke et al. (2018) [15], where there was no explicit mention of the amygdala, it is implied that there was a reduction in its activation after CBT. ACC activation was reduced and there was reduced activation in the dmPFC and dlPFC, which indicates better efficiency over emotional regulation and less need for cognitive efforts to handle stimuli. Both studies seemed to partially support the dual-route model, although there needs to be an emphasis on areas involved in interoception, such as the insula or ACC, and internal signals monitoring, which expands the traditional dual-route model.

Overall, the findings partially support the dual-route model in the case of CBT for panic disorder and specific phobias, where consistent reductions in limbic activation (e.g., amygdala) and adjustments in the prefrontal regions responsible for emotional regulation were observed. However, in SAD, the results are more varied. While there is evidence for reduced limbic reactivity, the increased prefrontal connectivity may indicate a stronger engagement of emotional control pathways, suggesting that the dual-route model may require adaptation depending on the specific disorder.

Across all disorders, the amygdala and ACC activity reductions reflect a shared mechanism of improved fear and emotion regulation following CBT. These findings align well with the dual-route model framework. Nevertheless, differences in the precuneus and PFC activities highlight the need for disorder-specific adaptations of the dual-route model. For example, changes in the precuneus activity in specific phobias and SAD suggest unique pathways for self-referential and emotional processing that may not align directly with the dual-route model. Taking this diversity into account, there is a need for the study of the dual-route model and its possible expansion to other areas.

From this scoping review, the main conclusion that can be extracted is that the dual-route model does not necessarily apply to all anxiety disorders, and more areas are involved depending on which disorder is being studied. On the other hand, for specific phobias, while it has been widely accepted that CBT’s effectiveness relies on this dual-route model, the data imply that the dual-route model may not be enough to explain why CBT works, and there are several areas that should be studied to further complete this model and expand it, such as the precuneus, the activation of which was consistently shown to be affected after CBT.

While the findings of this review support the dual-route model as a key framework for understanding CBT’s neurobiological effects, they also highlight areas where the model may need refinement or expansion. Specifically, differences in the precuneus activity across anxiety disorders suggest that additional mechanisms beyond prefrontal–limbic regulation might be at play.

One potential alternative mechanism is neuroplasticity, as repeated cognitive and behavioral interventions in CBT may induce structural and functional changes over time, particularly in regions involved in self-referential processing and emotional regulation. Additionally, predictive coding models propose that anxiety is maintained by maladaptive anticipatory responses, and CBT may work by recalibrating these expectations, reducing excessive prediction errors in the brain’s fear-processing circuits. Finally, large-scale networks, such as the salience network, default mode network (DMN), and executive control network, play crucial roles in emotion regulation and cognitive control, and their interactions may be key in explaining the variability in neural responses observed across different anxiety disorders.

These alternative explanations do not contradict the dual-route model but rather suggest that a more integrative framework may be needed. Future research should explore how these mechanisms interact with the traditional prefrontal–limbic regulation model to provide a more comprehensive understanding of CBT’s effects at the neural level. The dual-route model remains a valuable framework for understanding CBT’s neurobiological effects. However, future research should investigate the role of self-referential processing and variability in regulatory regional activation to refine the model for different anxiety disorders.

### 4.4. Limitations

The results from the research conducted indicate that since 2018, 12 studies have been focused on finding the reasons why CBT is effective for anxiety disorders at the brain activation level, which, in itself, presents a limitation on extracting conclusions.

Another potential limitation of this review was the heterogeneity in neuroimaging methodologies across the included studies. Variations in the fMRI acquisition parameters, preprocessing pipelines, and statistical analyses may have contributed to the differences in the reported findings. Additionally, some studies used task-based fMRI, while others relied on resting-state functional connectivity, which could lead to differences in the interpretation of the CBT-related changes in brain activity. Such methodological differences could introduce the variability in the observed neurobiological effects of CBT, potentially influencing the generalizability of the dual-route model across anxiety disorders. Future research should aim for a greater standardization of neuroimaging protocols, including uniform task paradigms and data-processing techniques, to improve cross-study comparability and enhance the robustness of findings related to CBT-induced neural changes.

Another limitation of this review was the variability in the demographic characteristics across studies, including differences in age, gender distribution, and medication use. While this scoping review focused on mapping previous research rather than directly comparing individual study results, these demographic factors may influence the neural responses to CBT. For instance, age-related differences in neuroplasticity, the potential impact of hormonal variations on emotion regulation circuits, and the effects of psychotropic medication on brain activity could all contribute to variations in the reported findings. However, due to the nature of scoping reviews, we did not systematically analyze these factors across studies. Future systematic reviews and meta-analyses should consider exploring the role of demographic variability in modulating CBT-related neurobiological changes to refine our understanding of treatment mechanisms.

Another limitation of this review was the exclusion of studies that employed other neuroimaging techniques, such as EEG or PET. While fMRI provides crucial information on changes in brain activation and connectivity, techniques like EEG offer better temporal resolution of neural processes, and PET can provide insights into the metabolic changes underlying CBT effects. The absence of these approaches may limit a comprehensive understanding of the neurobiological mechanisms of treatment. As a result, future reviews should consider a broader integration of methodologies to obtain a more complete picture of CBT’s effects on the brain.

Finally, another limitation of this review was the exclusion of studies that did not report symptom improvement following CBT. This criterion was applied to ensure that the identified neuroimaging changes were directly associated with the treatment efficacy. However, this approach may introduce survivorship bias, as it does not account for the potential neurobiological variations in non-responders. Future research should incorporate studies with mixed or null outcomes to provide a more comprehensive understanding of CBT’s neural effects, including factors that influence treatment resistance.

## Figures and Tables

**Table 1:** Results.

Results	Participants	Disorder	Author
A decreased amplitude of low-frequency fluctuation (ALFF) was observed in the right precuneus. A reduced degree centrality (DC) in the left precuneus and left middle temporal gyrus, as well as an increased DC in the right putamen.	15 patients and 19 healthy controls	SAD	Yuan et al. (2018) [14]
Reductions in the amygdala, left middle-superior temporal gyrus, and dorsomedial (dmPFC) and dorsolateral prefrontal cortex (dlPFC). A decreased connectivity between the right amygdala and left precuneus/posterior cingulate cortex during emotion regulation tasks.	28 participants: 14 in treatment group and 14 in waiting group	Panic disorder	Reinecke et al. (2018) [15]
Decreased activation in the left insula and anterior cingulate cortex during self-referential processing. A stronger positive connectivity between the amygdala and FFA during self-referential processing in participants with greater reductions in social anxiety severity (measured by the LSAS). A decreased connectivity between the amygdala and insula in the treatment group but increased in the waitlist group.	64 participants: 17 participants in the CBT group, 20 in the ACT group, 14 on the waitlist, and 13 healthy controls	SAD	Brown et al. (2019) [16]
Reduced amygdala reactivity. At the 6-month follow-up, initial reductions in the amygdala activation still predicted avoidance. Decreased activation in the anterior insula, dorsal hippocampus, supplementary motor area, and visual cortex during repeated exposure to spider images.	45 participants	Specific phobia	Björkstrand et al. (2020) [17]
Attenuation of neural activation in the anterior cingulate cortex for the processing of panic-trigger/panic-symptom word pairs.	42 patients and 52 healthy controls	Panic disorder	Yang et al. (2020) [18]
Negative changes in the amygdala connectivity with regulatory brain regions (e.g., dorsomedial prefrontal cortex [dmPFC] and dorsal anterior cingulate cortex [dACC]).	23 participants: 11 patients in the CBT group and 12 in the ACT group	SAD	Sandman et al. (2020) [19]
Increased activation in the precuneus, and reduced activation in fear-related regions, such as the thalamus and visual cortex (e.g., the calcarine gyrus).	32 participants: 16 patients and 16 healthy controls	Specific phobia	Viña et al. (2020) [20]
Reduced activity in the thalamus, fusiform gyrus, and dorsolateral prefrontal cortex after the treatment. The amygdala activity was reduced but still present.	31 participants: 17 received CBT with real images and 14 received CBT with VR	Specific phobia	Álvarez-Pérez et al. (2021) [21]
Increased activation in the posterior cingulate cortex/precuneus, lingual gyrus, inferior temporal gyrus, precentral gyrus, and postcentral gyrus during positive self-referential processing. There was also enhanced activation in the middle occipital gyrus, parahippocampus, Rolandic operculum, superior frontal gyrus, and caudate nucleus during negative self-referential processing.	21 patients and 22 healthy controls	SAD	Hur et al. (2021) [22]
Exposure-only condition (E): greater activation in fear-related regions: amygdala, insula, anterior and middle cingulate cortex, and ventromedial prefrontal cortex. Increased activity in sensory-perceptive and motor areas: postcentral gyrus, precentral gyrus, and superior occipital cortex. Self-verbalization condition (S): activation of top-down regulatory areas: inferior frontal gyrus (pars opercularis and pars triangularis). Breathing condition (B): reduced activation in fear-related areas.	30 participants: 9 participants received self-verbalization training (S), 10 participants received breathing training (B), and 11 participants experienced the exposure-only (E) condition	Specific phobia	Fumero et al. (2022) [23]
Decreased nodal efficiency in the left inferior frontal gyrus (language processing circuits) and left Heschl’s gyrus (auditory language comprehension) and increased degree centrality in the right calcarine sulcus (visual processing) and left dorsolateral prefrontal cortex (reduced overactivation, suggesting improved cognitive control).	52 participants: 24 patients and 28 in the control group	SAD	Kim et al. (2022) [24]
Decrease in activity in the amygdala, anterior cingulate cortex, precuneus, insula, and parietal cortex. The ACC activation decrease correlated with reductions in dental fear.	17 patients and 17 healthy controls	Specific phobia	Wannemueller et al. (2024) [25]

**Table 2:** Number of articles by disorder.

Articles	Disorder
5	Specific phobias
2	Panic disorder
4	Social anxiety disorder

**Table 3:** Brain region activation in specific phobias.

	Studies That Reported Hyperactivation Pre-CBT	Studies That Reported Reduced Activation Post-CBT	Studies That Reported Increased Activation Post-CBT
Amygdala	4	4	1
Anterior cingulate cortex	1	1	0
Ventromedial prefrontal cortex	1	1	1
Dorsolateral prefrontal cortex	2	1	0
Thalamus	1	1	0

**Table 4:** Brain region activation in social anxiety disorder.

	Studies That Reported Hyperactivation Pre-CBT	Studies That Reported Reduced Activation Post-CBT	Studies That Reported Increased Activation Post-CBT
Amygdala	3	3	0
Anterior cingulate cortex	1	1	1
Ventromedial prefrontal cortex	0	0	0
Dorsolateral prefrontal cortex	0	0	2
Thalamus	0	0	0

**Table 5:** Brain region activation in panic disorder.

	Studies That Reported Hyperactivation Pre-CBT	Studies That Reported Reduced Activation Post-CBT	Studies That Reported Increased Activation Post-CBT
Amygdala	1	1	0
Anterior cingulate cortex	1	1	0
Ventromedial prefrontal cortex	0	0	0
Dorsolateral prefrontal cortex	1	1	0
Thalamus	0	0	0

## Data Availability

Data are contained within the article.

## Abbreviations

The following abbreviations are used in this manuscript:

CBT	Cognitive behavioral therapy
SAD	Social anxiety disorder
PRISMA	Preferred Reporting Items for Systematic Reviews
AAC	Anterior cingulate cortex
ALFF	Amplitude of low-frequency activity
DC	Degree centrality
LSAS	Liebowitz Social Anxiety Scale
FFA	Fusiform face area
EBA	Extrastriate body area
HC	Healthy control
dmPFC	Dorsomedial prefrontal cortex
dACC	Dorsal anterior cingulate cortex
dlPFC	Dorsolateral prefrontal cortex
